# Cross-Cultural Adaptation and Validation of the Simplified Chinese Version of the Lower Extremity Functional Scale

**DOI:** 10.1155/2020/1421429

**Published:** 2020-03-09

**Authors:** Chuanxin Zhang, Yaqun Liu, Shuai Yuan, Tianbo Yang, Yuan Gao, Chao Zhu, Zheru Ding

**Affiliations:** ^1^Department of Joint Surgery and Sports Medicine, Changzheng Hospital, Second Military Medical University, Shanghai 200003, China; ^2^Department of Rheumatology and Immunology, Changzheng Hospital, Second Military Medical University, Shanghai 200003, China; ^3^Department of Surgery and War Surgery, Changzheng Hospital, Second Military Medical University, Shanghai 200003, China; ^4^Department of Cardiothoracic Surgery, Changhai Hospital, Second Military Medical University, Shanghai 200003, China; ^5^Department of Nephrology, Changhai Hospital, Second Military Medical University, Shanghai 200433, China

## Abstract

**Purpose:**

The purpose of this study was to cross-culturally adapt and validate the Simplified Chinese version of the Lower Extremity Functional Scale (SC-LEFS).

**Methods:**

The original English version of the Lower Extremity Functional Scale was translated and cross-culturally adapted into Simplified Chinese according to international guidelines. The SC-LEFS and 36-Item Short-Form Health Survey were administered to 213 patients with lower extremity musculoskeletal disorders. Psychometric properties including internal consistency, test-retest reliability, content validity, and construct validity were tested.

**Results:**

There were no floor or ceiling effects for the SC-LEFS. The scale had high values for internal consistency (Cronbach *α* = 0.97) and test-retest reliability (intraclass correlation coefficient = 0.97). Corrected item-total correlations for every item ranged from 0.67 to 0.89. And the item-level content validity index (I-CVI) for each item ranged from 0.78 to 1.00. Principal component analysis revealed a one-factor structure. Nine of ten prior hypotheses were confirmed, which further supports good construct validity within the SC-LEFS.

**Conclusion:**

The SC-LEFS has high internal consistency, good test-retest reliability and content validity, convergent construct validity, and a one-factor structure. Thus, it could be regarded as a reliable and valid tool to assess activity limitations in Chinese patients with lower extremity musculoskeletal disorders.

## 1. Introduction

Musculoskeletal disorders of the lower extremities, such as osteoarthritis and tendinitis, are common diseases that result in physical disability if not treated properly [1]. Clinicians need to evaluate the extent of activity limitations caused by these diseases to make effective and individual treatment decisions for patients [1, 2].

Until now, there has been no Simplified Chinese version of a questionnaire for evaluating activity limitations caused by lower extremity musculoskeletal disorders. Clinicians and researchers in mainland China use a variety of general health status questionnaires, such as the 36-Item Short-Form Health Survey (SF-36), or condition-specific questionnaires, such as the Arthritis Impact Measurement Scales, Western Ontario and McMaster Universities Osteoarthritis Index, Hip Osteoarthritis Outcome Score, and Knee Osteoarthritis Outcome Score [3–6]. These questionnaires were not specifically designed for patients with lower extremity musculoskeletal disorders and are usually not accurate in evaluating activity limitations [7–9].

The Lower Extremity Functional Scale (LEFS) is a 20-item region-specific outcome questionnaire designed to evaluate the activity limitations of patients with lower extremity musculoskeletal disorders [10]. There are 20 items on the LEFS, and each item scores from 0 to 4 on a Likert scale, producing an overall score ranging from 0 to 80. A lower score indicates more severe activity limitations [10]. The developers of the LEFS demonstrated that this scale had good reliability and validity [10]. Moreover, the LEFS has been successfully translated, cross-culturally adapted, and validated in a variety of countries, including Italy [11], the Netherlands [12], Spain [13], Greece [14], Brazil [15], Saudi Arabia [16], and Iran [17]. Studies in different cultures also demonstrated that the LEFS had good reliability and validity.

However, a Simplified Chinese version of the LEFS is not currently available, and it is inconvenient for clinicians from mainland China to assess the activity limitations of patients with lower extremity musculoskeletal disorders. Therefore, this study is aimed at translating the original English version of the LEFS into Simplified Chinese, making cross-cultural adaptations, and evaluating the psychometric properties (internal consistency, test-retest reliability, content validity, and construct validity) of the Simplified Chinese version of the LEFS. We hypothesized that the SC-LEFS would have good internal consistency, test-retest reliability, and content validity and moderate or high correlations with physical function (PF), role physical (RP), body pain (BP), general health (GH), physical component scale (PCS), vitality (VT), social function (SF), role emotional (RE), mental health (MH), and mental component scale (MCS) from the SF-36.

## 2. Materials and Methods

### 2.1. Participants

Based on the recommended 1 : 10 ratio of the number of items to the number of participants [18], 213 patients with lower extremity musculoskeletal disorders, including osteoarthritis, muscle strain, ligament and meniscal injury, patellofemoral pain, fracture, and nonspecific sprain, were recruited from the Department of Joint Surgery, Changzheng Hospital, in Shanghai, China. Those under 18 years old, unable to understand and complete the questionnaires, or had cardiopulmonary or neurological comorbidities were excluded. After consenting to this research, all the participants were given a form for collection of demographic data, the SC-LEFS and the SF-36 for completion. All 213 participants completed the questionnaires independently. One week later, the LEFS was administered again to the participants and 69 of them returned the questionnaires. This study was approved by the Human Research Ethics Committee of Changzheng Hospital, and informed consent was obtained from all the participants involved.

### 2.2. The LEFS and SF-36

The LEFS is a 20-item region-specific outcome questionnaire designed to assess the activity limitations of patients with lower extremity musculoskeletal disorders [10]. Each item of the LEFS is scored on a 5-point scale ranging from 0 to 4, representing the extent of difficulty in performing different physical activities because of the lower extremity musculoskeletal disorders (0 = unable to perform the activity or extreme difficulty, 4 = no difficulty). Therefore, LEFS scores range from 0 to 80, with higher scores suggesting higher levels of functioning [10].

The SF-36 is a 36-item questionnaire designed to assess the general health status of patients [3]. It consists of 8 subscales, including physical function (PF), role physical (RP), body pain (BP), general health (GH), vitality (VT), social function (SF), role emotional (RE), and mental health (MH), which are then aggregated into two summary scales, namely, the physical component scale (PCS) and mental component scale (MCS). Each subscale score ranges from 0 to 100, with higher scores suggesting better health status. The Chinese version of the SF-36 has been validated with good reliability and validity [3].

### 2.3. Translation and Cross-Cultural Adaptation of the LEFS

The original English version of the LEFS was translated into Simplified Chinese and cross-culturally adapted based on current guidelines [18, 19]. Two bilingual translators, whose first language was Chinese, translated the English version into Simplified Chinese independently. One translator, a researcher of this study, was acquainted with the purpose of translation. The other translator, an expert majoring in English, was completely blinded to this study. The two independent forward translations were integrated and then synthesized into one version. Next, a back translation was performed by two independent translators whose first language was English. Then, an expert committee consisting of orthopaedists, rehabilitation physicians, health professionals, statisticians, and a linguist was established to compare all the translated versions with the original English version. Finally, a single Simplified Chinese version was developed by consensus.

A pretest of the Simplified Chinese version of the LEFS was conducted on 38 patients with lower extremity musculoskeletal disorders. Every patient was asked to complete the scale and to identify whether there existed any confusion or difficulty in comprehending each item. Their answers and suggestions were recorded by the expert committee. After proper modifications, the final version of the LEFS in Simplified Chinese was developed.

### 2.4. Statistical Analysis

Descriptive statistics were utilized to summarize all the demographic characteristics. The psychometric properties of the SC-LEFS, including floor and ceiling effects, internal consistency, test-retest reliability, minimally detectable change, content validity, and construct validity, were examined with the IBM Statistic Package for Social Science 25.0 (IBM Corp., Armonk, NY) [18–20]. For all analyses, the level of statistical significance was set at 0.05.

Floor and ceiling effects were determined by computing the percentage of participants who achieved the lowest (0) or highest (80) score. The effects were thought to be present if over 15% of the participants obtained the lowest or highest score [21].

Internal consistency reflects the homogeneity of a questionnaire, and it was assessed with Cronbach's alpha. Internal consistency is considered good if Cronbach's alpha is between 0.7 and 0.9 [22, 23].

Test-retest reliability refers to the consistency of a questionnaire over time. It was assessed with intraclass correlation coefficients (ICCs; a two-way ANOVA with absolute agreement), which range from 0 to 1 [24, 25]. A higher score indicates a higher level of reliability, and an ICC exceeding 0.9 is considered good. Absolute reliability was assessed with the standard error of measurement (SEM), which represents the measurement error expressed in the same unit as the original measurement. SEM was computed according to the following formula: SEM = SD∗[√1 − ICC], in which SD is the standard deviation and ICC is the intraclass correlation coefficient [24].

The minimal detectable change (MDC) at the 95% confidence level was also computed from the SEM and indicated the amount of change that is required to be confident of a clinically important change and not a change due to error variation. The MDC_95_ was computed according to the following formula: MDC_95_ = SEM∗1.96∗√2 [25].

Content validity is used to examine whether the items in the questionnaire could evaluate the concept sufficiently and adequately [23]. All the participants were asked if they had any difficulties or confusion in understanding each item. Each item was analysed to test whether it could measure the same property after being translated into Simplified Chinese and cross-culturally adapted for patients in mainland China. To test the content validity index, the relevance of each item of the final version of the questionnaire was evaluated by a panel of 9 experts, including 3 orthopaedists, 3 rehabilitation physicians, and 3 nursing experts. Each item was scored by every expert on a 4-point Likert scale (1 = not relevant, 2 = somewhat relevant, 3 = quite relevant, and 4 = highly relevant) [26]. The item-level CVI (I-CVI) was calculated by counting the number of experts who scored “3” or “4” and then dividing the number by the total number of experts who answered the question [26]. Content validity was considered as good if the I-CVI value is above 0.74 [27].

Construct validity reflects the degree to which a construct measures the intended concept [28]. The construct validity of the LEFS was examined by factor analysis and hypothesis testing. To explore the potential structure of the SC-LEFS, an exploratory factor analysis (EFA) was performed by principal component analysis (PCA) with varimax rotation. Hypothesis testing was performed by examining the correlation between the LEFS scores and SF-36 scores using Pearson's correlation coefficient. Correlation coefficients less than 0.3, between 0.3 and 0.6, and over 0.6 were considered low, moderate, and high, respectively [28]. The SF-36 has been validated in mainland China, and it is believed that the PF and BP subscales are the most relevant concepts for lower extremity musculoskeletal disorders, while mental health is the least relevant concept [3, 11–15]. Therefore, we proposed the following hypotheses: the SC-LEFS should have moderate or high correlations with PF, RP, BP, GH, PCS, VT, SF, RE, MH, and MCS from the SF-36.

## 3. Results

### 3.1. Translation and Cross-Cultural Adaptation

The Simplified Chinese version of the LEFS was finally developed by the expert committee (see Supplementary Materials ([Supplementary-material supplementary-material-1])). All the items were confirmed to be easy, clear, and acceptable. One minor modification was performed on item 12 (walking a mile). The distance “a mile” was converted into the metric equivalent, i.e., 1.6 kilometres.

### 3.2. Descriptive Statistics

Two hundred and thirteen participants (101 males; 112 females) with lower extremity musculoskeletal disorders, aged 53.5 years (SD = 18), with a median disease duration of 8.3 years participated in this study (Table 1). The number of participants who had 0, 1, 2, 3, and 4 missing items was 173 (81.2%), 15 (7.0%), 11 (5.2%), 9 (4.2%), and 5 (2.3%), respectively (Table 1). The LEFS, PCS, and MCS scores averaged 45.1 (SD = 19.2), 37.5 (SD = 14.3), and 48.2 (SD = 20.1), respectively (Table 1).

### 3.3. Floor and Ceiling Effects

Only one participant (0.4%) scored the worst score (0), while no participant scored the best score, indicating that the SC-LEFS has no floor and ceiling effects.

### 3.4. Internal Consistency and Test-Retest Reliability

Cronbach's alpha for the SC-LEFS was 0.97. The item-deleted Cronbach's *α* of each item ranged from 0.971 to 0.973 (Table 2). The item-total correlation coefficients for the 20 items were all above 0.6 (Table 2). Therefore, the internal consistency of the SC-LEFS was considered good. The ICC value was 0.97 (95% CI, 0.95-0.98), suggesting that the SC-LEFS has good test-retest reliability (Table 3). The SEM and MDC_95_ were 3.3 and 9.2, respectively. Thus, the SC-LEFS had good reliability (Table 3).

### 3.5. Content Validity

The translation and cross-cultural adaptation processes were examined by the expert committee, and the final Simplified Chinese version of the LEFS was developed successfully. All the participants finished the questionnaire independently with no difficulties or confusion. And all 20 items yielded I-CVI scores above the recommended limit (I − CVI ≥ 0.74) (Table 3). Thus, the SC-LEFS has good content validity, and no item should be eliminated.

### 3.6. Construct Validity

Structural construct validity and convergent construct validity were examined by PCA and hypothesis testing, respectively.

PCA suggested a one-factor structure. This factor had an eigenvalue of 13.44, explaining 67% of the total variance (Table 4). All the items had loadings over 0.7 on the first factor (Table 4). The value of factor loadings ranged from 0.705 for item 15 to 0.909 for item 12 (Table 4). The Kaiser–Meyer–Olkin Measure of Sampling Adequacy (KMO) was utilized to assess the adequacy of the sample used in the study to perform factor analysis. The statistical value of the KMO was 0.92, implying that the sample size used in the factor analysis was sufficient.

The SC-LEFS was found to have a high correlation with the PCS (*r* = 0.70, *P* < 0.01) and a weak correlation with the MCS (*r* = 0.39, *P* < 0.05), as expected (Table 5). Regarding the correlations between the SC-LEFS and the 8 subscales of the SF-36, the SC-LEFS had a strong correlation with PF (*r* = 0.85, *P* < 0.01) and moderate correlations with RP (*r* = 0.40, *P* < 0.01), BP (*r* = 0.51, *P* < 0.01), GH (*r* = 0.45, *P* < 0.01), SF (*r* = 0.41, *P* < 0.01), VT (*r* = 0.34, *P* < 0.05), and MH (*r* = 0.38, *P* < 0.05) (Table 5). However, there was no significant correlation between the SC-LEFS and RE (*r* = 0.12, *P* > 0.05) (Table 5). Thus, nine of ten hypotheses were verified, and the SC-LEFS was thought to have good convergent construct validity.

## 4. Discussion

The objectives of this study were to cross-culturally adapt the original English version of the LEFS into Simplified Chinese and to evaluate its psychometric properties. The SC-LEFS for Chinese patients was adapted based on international guidelines [18, 19]. There were no difficulties in translating the questionnaire, and the back translation version was in agreement with the original version. The only minor modification was the change in the metric unit for walking distance. The distance “a mile” in item 12 was converted into the metric equivalent, i.e., 1.6 kilometres (km) because Chinese people were more familiar with this metric unit. All the participants finished the questionnaire independently with no difficulties or confusion. And all the 20 items yielded I-CVI scores above the recommended limit. Thus, the SC-LEFS has good content validity, and no item should be eliminated.

Cronbach's *α* coefficient for SC-LEFS was 0.97, indicating good internal consistency [20]. Compared with other versions of the LEFS, this result is slightly better than that in the original version (0.96) [10], the Italian version (0.96) [11], the Arabic version (0.95) [16], and the Dutch version (0.94) [12]. The ICC for the LEFS was 0.97 (95% CI: 0.95-0.98), indicating good test-retest reliability. This result is also better than that in other versions, such as the original version (0.86) [10] and the Italian version (0.89) [11]. Corrected item-total correlation measures the degree to which each item correlates with the total scale. All 20 items had corrected item-total correlations above 0.6, indicating that each item in the scale correlates well with the total scale and that all of them could help to discriminate between patients. Therefore, the Chinese version could be considered a reliable tool.

The SEM of the SC-LEFS was 3.3, while the MDC_95_ was 9.2. When presented as a percentage of the average SC-LEFS score, the SEM was 7.3%, while the MDC_95_ was 20.4%. Based on the above results, the measurement error of the SC-LEFS and the MDC is considered to be clinically applicable. The value of the MDC (9.2) for the SC-LEFS is very close to that reported for the original version (9.0) [10] and the Arabic version (9.8) [16], and it ranges from 7 to 12 scale points in most of the translated versions. However, the MDCs reported for the Spanish version and Italian version were 2.18 and 2.4, respectively, which differ significantly from those for most versions of the LEFS [11, 13].

Factor analysis was performed to explore the structure of the SC-LEFS. We hypothesized that the SC-LEFS would have a one-factor structure according to previous studies. Factor analysis revealed one major factor within the SC-LEFS. The major factor explained 67.2% of the total variance, and all the items in the SC-LEFS loaded significantly on this factor. The results of factor analysis indicate that the SC-LEFS has good construct validity, and the scale mainly measures activity limitations because of lower extremity musculoskeletal disorders. The underlying one-factor structure in the SC-LEFS is consistent with that in other versions, such as the original [10], Dutch [12], Spanish [13], and Arabic [16] versions.

Additionally, the results of the hypothesis testing also support the good construct validity of the SC-LEFS. Nine of ten hypotheses were confirmed by examining the internal correlations between the SC-LEFS and the SF-36. Compared with the original English version of the LEFS, the SC-LEFS demonstrated similar correlations with the SF-36. We observed that the SC-LEFS had higher correlations with the SF-36 PCS than with the SF-36 MCS. For the eight subscales of the SF-36, the SC-LEFS had high correlations with PF, moderate correlations with RP, BP, GH, and SF, and weak correlations with VT and MH. One result that we did not expect was that the SC-LEFS did not correlate with RE, while the original LEFS showed a low correlation with RM. We speculated that the difference might result from the different disease severity of the participants involved in the different studies [29]. The disease severity of participants in this study was lower than that in the previous samples, and thus, activity limitations of the participants did not lead to significant changes in role emotional [30].

The SC-LEFS had no floor or ceiling effects, which further confirmed its validity. Thus, the validation process demonstrated that the SC-LEFS had good reliability and validity, which are comparable with the original LEFS version and another Asian version (the Arabic version) [16].

However, there are several limitations in the study. First, the mean age of all the participants was 53.5 years, and the applicability of the SC-LEFS for young people needs to be investigated in future studies. Second, the sensitivity to change was not assessed as a consequence of the lack of long-term follow-up or as a gold standard for changes in patients' general health status [31, 32]. Thirdly, the value of Cronbach's alpha more than 0.9 indicated that there was item redundancy in the scale. However, due to the large sample size, a wide scale (5-point scale), and a more heterogeneous population in this study, the figure might inflate [33, 34]. And the value of Cronbach's alpha in our study was comparable to the findings of the original English version and previous validation studies (Cronbach's alpha ranging from 0.94 to 0.989) [10–13, 15–17].

## 5. Conclusion

In summary, the original English version of the LEFS was successfully translated and adapted into a Simplified Chinese version. The SC-LEFS has high internal consistency, good test-retest reliability and content validity, convergent construct validity, and a one-factor structure. Thus, the SC-LEFS could be regarded as a reliable and valid tool to assess activity limitations in Chinese patients with lower extremity musculoskeletal disorders.

## Figures and Tables

**Table 1:** Characteristics of patients with lower extremity musculoskeletal disorders (*N* = 213).

Characteristics	Mean (SD) or median	*N* (%)
Age (yr), mean (SD)	53.5 (18.0)	
Sex		
Male		101 (47.4%)
Female		112 (52.6%)
Height (cm), mean (SD)	169.5 (10.2)	
Weight (kg), mean (SD)	71.4 (19.5)	
Disease duration (yr), median	8.3	
Marital status		
Single		50 (23.5%)
Married		163 (76.5%)
Education		
Elementary		49 (23.0%)
Secondary		108 (50.7%)
University		56 (26.3%)
Side involved, right/left (*n*)		98/115
Types of musculoskeletal dysfunction		
Osteoarthritis		45 (21.2%)
Muscle strain		38 (17.8%)
Ligament injury		32 (15.0%)
Meniscal injury		27 (12.6%)
Patellofemoral pain		19 (8.9%)
Fracture		21 (9.9%)
Nonspecific sprain		31 (14.6%)
Site		
Hip and thigh		75 (23.0%)
Knee and leg		97 (23.0%)
Ankle and foot		41 (23.0%)
Surgery (yes/no)		31/182
LEFS score, mean (SD)	45.1 (19.2)	
PCS, mean (SD)	37.5 (14.3)	
MCS, mean (SD)	48.2 (20.1)	
Numbers of missing items		
0		173 (81.2%)
1		15 (7.0%)
2		11 (5.2%)
3		9 (4.2%)
4		5 (2.3%)

SD: standard deviation; *N*: number; yr: year; LEFS: Lower Extremity Functional Scale; PCS: physical component scale; MCS: mental component scale.

**Table 2:** Corrected item-total correlations, Cronbach's alpha if the item was deleted, and I-CVI for every item.

Item	Corrected item-total correlation	Cronbach's alpha if the item was deleted	I-CVI
1	0.863	0.971	0.89
2	0.674	0.973	0.89
3	0.771	0.972	1.00
4	0.826	0.972	0.78
5	0.698	0.973	1.00
6	0.861	0.971	1.00
7	0.757	0.972	0.89
8	0.818	0.972	1.00
9	0.821	0.972	0.78
10	0.866	0.971	0.89
11	0.889	0.971	0.89
12	0.844	0.971	0.78
13	0.895	0.971	0.78
14	0.862	0.971	0.89
15	0.678	0.973	1.00
16	0.79	0.972	0.89
17	0.809	0.972	0.89
18	0.759	0.972	1.00
19	0.741	0.972	0.89
20	0.697	0.973	0.89

I-CVI: item-level content validity index.

**Table 3:** Average score, ICC, SEM, and MDC_95_ on the SC-LEFS in patients who were retested.

Questionnaire	Test (*n* = 69)	Retest (*n* = 69)	ICC (95% CI)	SEM	MDC_95_
SC-LEFS	44.9 (19.1)	45.7 (20.6)	0.97 (0.95-0.98)	3.3	9.2

ICC: intraclass correlation coefficient; SEM: standard error of measurement; MDC_95_: minimally detectable change at the 95% confidence level; SC-LEFS: Simplified Chinese version of the Lower Extremity Functional Scale.

**Table 4:** Factor structure for the SC-LEFS.

Factor	Initial eigenvalues			Item	Factor loading
	Total	% of variance	Cumulative %		
1	13.44	67.201	67.201	1	0.879
2	0.947	9.734	76.935	2	0.706
3	0.848	5.24	82.175	3	0.792
4	0.654	3.272	85.447	4	0.847
5	0.556	2.778	88.225	5	0.724
6	0.479	2.394	90.619	6	0.878
7	0.441	2.207	92.826	7	0.779
8	0.343	1.713	94.539	8	0.839
9	0.194	0.969	95.508	9	0.842
10	0.172	0.862	96.37	10	0.885
11	0.157	0.787	97.157	11	0.9
12	0.135	0.676	97.833	12	0.861
13	0.108	0.54	98.373	13	0.909
14	0.092	0.462	98.835	14	0.876
15	0.07	0.352	99.186	15	0.705
16	0.05	0.252	99.439	16	0.819
17	0.04	0.199	99.637	17	0.828
18	0.034	0.172	99.809	18	0.782
19	0.02	0.102	99.911	19	0.769
20	0.018	0.089	100	20	0.725

SC-LEFS: Simplified Chinese version of the Lower Extremity Functional Scale.

**Table 5:** Correlations between the scores of the SC-LEFS and the SF-36 subscales.

SF-36 subscales	*r* coefficient	*P* value
Physical function	0.85	<0.01
Physical role	0.40	<0.01
Body pain	0.51	<0.01
General health	0.45	<0.01
Vitality	0.34	<0.05
Social function	0.41	<0.01
Role emotional	0.12	>0.05
Mental health	0.38	<0.05
Physical component scale	0.70	<0.01
Mental component scale	0.39	<0.01

SC-LEFS: Simplified Chinese version of the Lower Extremity Functional Scale; *r*: Pearson's correlation coefficient.

## Data Availability

The data used to support the findings of this study are available from the corresponding author upon request.

## Supplementary Materials

Supplementary Materials The Simplified Chinese version of the Lower Extremity Functional Scale (SC-LEFS).
