# Supplementary material for: Cross-Cultural Adaptation and Validation of the Simplified Chinese Version of the Lower Extremity Functional Scale
Source: Biomed Res Int. 2020 Mar 9;2020:1421429. doi: 10.1155/2020/1421429 (PMC7086410; doi:10.1155/2020/1421429)
Supplement: Supplementary Materials — The Simplified Chinese version of the Lower Extremity Functional Scale (SC-LEFS). [file 1421429.f1.pdf]

简体中文版下肢功能评估量表 (SC-LEFS)

您从事下列活动时，是否会有任何困难？请在每个问题后勾选一个最符合的选项。

活动	极度困难<br>或无法做到	非常困难	中等困难	有点困难	毫无困难
1、您平日任何的工作、家务或学校的活动	0	1	2	3	4
2、您平时的爱好、休闲娱乐或运动	0	1	2	3	4
3、进出浴缸	0	1	2	3	4
4、在房间中走动	0	1	2	3	4
5、穿鞋或穿袜子	0	1	2	3	4
6、蹲下	0	1	2	3	4
7、提举物品，如将地上的整袋杂货拿起来	0	1	2	3	4
8、在家里做一些轻松的劳动	0	1	2	3	4
9、在家里做一些重体力劳动	0	1	2	3	4
10、上车或下车	0	1	2	3	4
11、走两条街道的路	0	1	2	3	4
12、走 1.5 公里	0	1	2	3	4
13、上下十个台阶（约一段楼梯）	0	1	2	3	4
14、站一个小时	0	1	2	3	4
15、坐一个小时	0	1	2	3	4
16、在平坦的地面上跑步	0	1	2	3	4
17、在不平坦的地面上跑步	0	1	2	3	4
18、快跑时急速转弯	0	1	2	3	4
19、单脚跳	0	1	2	3	4
20、在床上翻身	0	1	2	3	4
